# Integrated Whole-Genome Sequencing and In Silico Characterization of *Salmonella* Cerro and Schwarzengrund from Brazil

**DOI:** 10.3390/genes16080880

**Published:** 2025-07-26

**Authors:** Nathaly Barros Nunes, Vinicius Silva Castro, Adelino da Cunha-Neto, Fernanda Tavares Carvalho, Ricardo César Tavares Carvalho, Eduardo Eustáquio de Souza Figueiredo

**Affiliations:** 1 Faculty of Agronomy and Zootechnics, Federal University of Mato Grosso (UFMT), Cuiabá 78060-900, MT, Brazil; viniciuscastro06@gmail.com; 2 Faculty of Nutrition, Federal University of Mato Grosso (UFMT), Cuiabá 78060-900, MT, Brazil; adelino.neto@ufmt.br; 3 Program in Animal Bioscience, University of Cuiabá (UNIC), Cuiabá 78065-900, MT, Brazil; fefetavares_carvalho@hotmail.com (F.T.C.); ricardo_carvalho88@hotmail.com (R.C.T.C.)

**Keywords:** whole-genome sequencing, WGS, *Salmonella* Cerro, *Salmonella* Schwarzengrund, pathogenicity, bacterial virulence, cheese, beef

## Abstract

Background: *Salmonella* is a bacterium that causes foodborne infections. This study characterized two strains isolated from cheese and beef in Brazil using whole-genome sequencing (WGS). Objectives: We evaluated their antimicrobial resistance profiles, virulence factors, plasmid content, serotypes and phylogenetic relationships. Methods: DNA was extracted and sequenced on the NovaSeq 6000 platform; the pangenome was assembled using the Roary tool; and the phylogenetic tree was constructed via IQ-TREE. Results and Discussion: For contextualization and comparison, 3493 *Salmonella* genomes of Brazilian origin from NCBI were analyzed. In our isolates, both strains carried the *aac(6′)-Iaa_1* gene, while only Schwarzengrund harbored the *qnrB19_1* gene and the *Col440I_1* plasmid. Cerro presented the islands SPI-1, SPI-2, SPI-3, SPI-4, SPI-5 and SPI-9, while Schwarzengrund also possessed SPI-13 and SPI-14. Upon comparison with other Brazilian genomes, we observed that Cerro and Schwarzengrund represented only 0.40% and 2.03% of the national database, respectively. Furthermore, they revealed that Schwarzengrund presented higher levels of antimicrobial resistance, a finding supported by the higher frequency of plasmids in this serovar. Furthermore, national data corroborated our findings that SPI-13 and SPI-14 were absent in Cerro. A virulence analysis revealed distinct profiles: the *cdtB* and *pltABC* genes were present in the Schwarzengrund isolates, while the *sseK* and *tldE*1 family genes were exclusive to Cerro. The results indicated that the sequenced strains have pathogenic potential but exhibit low levels of antimicrobial resistance compared to national data. The greater diversity of SPIs in Schwarzengrund explains their prevalence and higher virulence potential. Conclusions: Finally, the serovars exhibit distinct virulence profiles, which results in different clinical outcomes.

## 1. Introduction

Foodborne illnesses caused by pathogens represent a major public health concern worldwide [1]. Among these, salmonellosis stands out as one of the most significant foodborne bacterial zoonoses [2]. The genus *Salmonella* comprises two species: *Salmonella bongori* and *Salmonella enterica* [3]. *S. enterica* currently includes approximately 2600 serotypes [4].

*Salmonella* has been widely reported in cattle [1] and dairy cows [2]. Its presence in bovine can lead to the contamination of milk or meat through various environmental sources on the farm, including other animals, rodents, wildlife, insects, water, resulting in both direct and indirect infection of humans and other animals [3]. *Salmonella* Cerro has been detected in dairy herds as an asymptomatic carrier, with fecal shedding observed in clinically healthy animals [4]. Although this serotype is rarely associated with human illness [5,6], it has emerged in recent years due to its increasing prevalence among dairy cattle [6].

*Salmonella* Schwarzengrund is frequently associated with human infections [7] and is also commonly reported in farm animals, food products and agricultural environments, where it has been linked to antimicrobial-resistant infections in humans [8]. In many countries, *S.* Schwarzengrund ranks among the top 15 *Salmonella* serotypes isolated from humans, food sources and animals [9].

Data from the United States Department of Agriculture [10] indicate that the global cattle herd is expected to reach approximately 914 million head in 2025. This report also describes the global distribution of cattle, with Brazil ranking second place in herd size, behind only India among the seven regions analyzed. Between 2014 and 2024, Brazilian beef production increased by 21.88% (in thousand tons). According to the CiCarne Yearbook of the beef production chain, published in March 2025, Brazil is recognized for its high food productivity, particularly in animal-based foods, and it is the world’s largest exporter of the most widely consumed meat [11].

The Midwest region is the largest producer of cattle in Brazil. In 2024, Mato Grosso state led all states in beef exports, shipping 161,98 thousand tons abroad, with China and the United States as the main destinations [12]. Regarding milk production, recent data indicate that the Central-West region ranks third place among Brazilian regions, accounting for approximately 11.3% of the raw milk purchased by dairies. Despite these high levels of bovine productivity and exports, there are still limited studies on pathogenic bacteria in this sector, especially *Salmonella*.

In Brazil, several studies have utilized whole-genome sequencing (WGS) to investigate pathogenic bacteria, including various *Salmonella* serotypes [13,14,15,16,17]. Given the limitations of conventional serology, WGS has become a critical tool for characterizing foodborne pathogens and predicting genotypic antimicrobial resistance [18,19]. Insights into pathogen genomes are essential for disease prevention, management and treatment [20]. WGS enables the identification of resistance genes, virulence factors, mobile genetic elements and relevant mutations [21,22].

In a previous study, Cunha Neto et al. [23] analyzed 225 cheese samples from Brazilian dairies, and Muller et al. [24] examined 107 beef samples from various slaughterhouses in Mato Grosso, Brazil. In both cases, the isolates were phenotypically identified as *Salmonella* Schwarzengrund. However, it is well known that traditional isolation and serological methods have limited accuracy and may allow cross-reactions, which can hinder the complete characterization of bacterial strains. Therefore, the aim of this study was to sequence the first two genomes of *Salmonella* strains isolated from cheese and beef in Mato Grosso state, Brazil, to confirm their serovar designations and to genotypically characterize their antimicrobial resistance profile, virulence determinants and phylogenetic relationships in the context of other Brazilian *Salmonella* genomes.

## 2. Materials and Methods

### 2.1. Strain Recovery and DNA Extraction

The strains used in this study were previously identified as *Salmonella* Schwarzengrund in the studies by [23,24]. For DNA extraction, a single colony was resuspended in brain heart infusion (BHI) broth (KASVI^®^, laboratories Conda S.A, Madrid, Spain) and incubated overnight at 35 °C. Genomic DNA was then extracted and quantified using Qiagen DNeasy Blood and Tissue Kit (Qiagen, Hilden, Germany) and QUBIT 2.0 fluorometer (Invitrogen^®^, Carlsbad, CA, USA), respectively, following the manufacturer’s instructions.

### 2.2. Whole-Genome Sequencing and Data Processing

The sequencing library was prepared using the NEBNext Ultra II DNA library Prep Kit (New England Biolabs, Ipswich, MA, USA). Sequencing was performed on the Illumina NovaSeq 6000 platform (Illumina Inc., San Diego, CA, USA) by GenOne Biotechnologies (Rio de Janeiro, Brazil), achieving a minimum coverage depth of 100×.

The quality of raw sequencing reads was assessed using FastQC and further processed with fastp. De novo genome assembly was performed using Shovill, which employs SPAdes v.1.1.0 (https://github.com/tseemann/shovill) (accessed on 16 March 2025). The genome sequences generated in this study were deposited in the National Center for Biotechnology Information (NCBI) database under BioProject accession number PRJNA1154933.

In addition, all sequences available in the NCBI Pathogen Detection database (https://www.ncbi.nlm.nih.gov/pathogens/) (accessed on 15 March 2025) were downloaded after applying filters for *Salmonella* and Brazil. Thus, a total of 3493 sequences were retrieved on (17 March 2025) and subjected to serotyping using SeqSero2 tool v1.3.1 [25] based on the assembled contigs processed on a Linux Mint system. Of these, 71 assemblies were identified as *Salmonella enterica* serovar Schwarzengrund and 14 assemblies as *Salmonella enterica* serovar Cerro. Supplementary Materials Table S1 containing the accession numbers and associated metadata for each analyzed strain is available.

### 2.3. Genome Quality, Annotation, Virulence and Resistance Gene Determination

Genome quality was first assessed using QUAST (v. 5.3.0) with default parameters, generating PDF reports. In addition, NCBI assemblies were evaluated using the seqkit tool (v2.10.0), specifically the L50 metric. The L50 is defined as the minimum number of largest contigs whose combined length comprises at least 50% of the total genome assembly. In this study, the highest L50 value observed was 68, which nevertheless indicated that the assemblies from the Pathogen Detection database were of sufficient quality for downstream analyses. Supplementary Materials Table S1 is provided with N50, L50 and additional sequencing quality metrics.

Genome annotation was then performed with Prokka (1.14.6) [26], using GFF3 output format and setting a minimum contig size of 200 bp. For specialized gene annotation, including virulence genes, ABRicate v. 1.0.1 was employed with the VirulenceFinder Database [27]. Antimicrobial resistance genes were identified by running ABRIcate again, this time using the Resfinder database (updated on 22 March 2024) [28]. All ABRicate analyses were performed using the default parameters (80% coverage and identity). Pathogenicity islands were detected using SPIFinder 2.0, applying thresholds of 95% identity and 60% coverage [29,30,31]. For plasmid analysis, we used the PlasmidFinder (Version 2.1.6; Database version: 18 January 2023) [32]. All the analyses described above were carried out on a total of 84 strains retrieved from the NCBI Pathogen Detection database and the 2 newly sequenced strains in this study, totaling 86 strains.

### 2.4. Phylogenetic Tree Comparison

For a genome comparison, the GFF3 files generated by Prokka (as described above) were used to construct a pangenome from our set of 86 serotypes. The Roary tool (v3.13.0) [33] was run with a minimum sequence identity of 95% and a gene presence threshold of 99% of isolates to define the core genome. We then generated two figures: one showing the accessory genome (all non-core genes) and another depicting the pangenome based on core-genome genes.

After obtaining the accessory-genome alignment, a maximum-likelihood phylogenetic tree was constructed using IQ-TREE v2.4.0 [34], with ultrafast bootstrap analysis of 1.000 replicates. For the pangenome analysis, Single nucleotide polymorphisms (SNPs) were extracted from the core genome alignment using SNP, and a phylogenetic tree was constructed with FastTree 2.1.10 [35].

For the metadata of the tree figures, manual curation was performed by the authors, and the data were distributed into the following groups and depicted in a phylogenetic tree: carcass/meat/organ; clinical/fluids; environmental sources; fecal/swab sample; food/feed or others; type of isolation (clinical or environmental); serotype (Cerro or Schwarzengrund); presence or absence of genes in the pathogenicity islands; and resistance profile to some classes of antibiotics.

### 2.5. Data Processing and Visualization

The phylogenetic tree was visualized using the Microreact web platform, enabling interactive exploration of the tree alongside metadata [36]. Data processing and analysis, including the generation of the heatmap and grouped bar chart, were performed in Python (v. 3.10.11) using Pandas (1.5.2), Numpy (1.24.2), Matplotlib (3.6.3) and Seaborn (0.12.1).

Reports obtained from Resfinder, PlasmidFinder and VFDB were merged and converted into binary matrices (0 = absence; 1 = presence), considering only genes detected with an identity greater than 80%. For heatmap generation, these binary values were transformed into percentages (1 = 100%; 0 = 0%), and frequencies were calculated and visualized by color intensities. The heatmap illustrating the average percentage distribution of pathogenicity islands was generated with Seaborn to facilitate group comparisons. For antimicrobial resistance gene analysis, the Resfinder results were processed and visualized as grouped bar charts using Matplotlib, highlighting the incidence of resistance genes across serotypes.

## 3. Results

### 3.1. Serovar Determination and Sequence Classification

*Salmonella*_R5748 was identified as serovar Cerro, while *Salmonella*_R10633 was classified as Schwarzengrund. To assess the prevalence of these serovars in Brazil, we queried the Pathogen Detection database (https://www.ncbi.nlm.nih.gov/pathogens/) (accessed on 21 April 2025) for additional *Salmonella* sequences of Brazilian origin. Serotype assignments were performed using SeqSero2 [25]. In total, 3493 sequences were retrieved from NCBI; of these, 71 (2,03%) were identified as Schwarzengrund and 14 (0.40%) as Cerro.

Based on the metadata, the distribution of Schwarzengrund isolates was as follows: 49.3% (35/71) were obtained from carcass/meat/organs; 36.6% (26/71) from environmental sources; 5.6% (4/71) from food or animal feed (including *Salmonella*_R10633); 4.2% (3/71) from fecal samples or anal junction swabs; 2.8% (2/71) from other or undetermined sources; and 1.4% (1/71) from clinical specimens. For Cerro, 28.6% (4/14) of the isolates were obtained from carcass/meat/organs; 21.4% (3/14) from environmental sources; 21.4% (3/14) from fecal/swab samples; 14.3% (2/14) from food or animal feed; and 14.3% (2/14) from other/undetermined sources.

Notably, both [23,24] identified these isolates as *Salmonella* Schwarzengrund using conventional serology. This discrepancy between in vitro serological results and in silico genomic analysis represents the first major inconsistency observed in this study.

### 3.2. Detection of Resistance Genes and Plasmids in Salmonella Cerro and Schwarzengrund

RESfinder and PlasmidFinder were applied to the WGS data to identify resistance genes and plasmids in *S.* Cerro (*Salmonella*_R5748) and *S.* Schwarzengrund (*Salmonella*_R10633). Both strains harbored the *aac(6′)-Iaa_1*, which confers resistance to the aminoglycosides amikacin (AMI) and tobramycin (TOB). In the *S.* Schwarzengrund isolate, the *qnr*B19_1 gene was also identified, encoding resistance to the fluoroquinolone ciprofloxacin (CIP). Thus, resistance in these sequenced strains was associated with the aminoglycoside and quinolone classes, although few genes were identified. No plasmid was detected in the *S.* Cerro (*Salmonella*_R5748), whereas *S.* Schwarzengrund (*Salmonella*_R10633) harbored the *Col440I_1* plasmid (Table 1).

**Figure 1:**
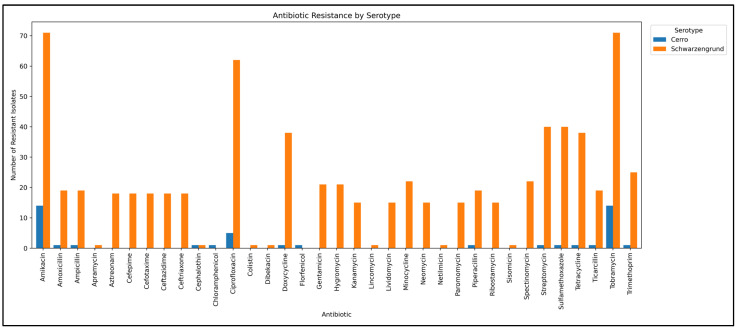
Frequency (%) of antimicrobial resistance genes in *S.* Cerro and *S.* Schwarzengrund isolates. Legend: Bar graph showing the resistance profile of Cerro (blue) and Schwarzengrund (orange). The x-axis indicates the antibiotics, and the y-axis indicates the frequency of antibiotic resistance.

To compare the resistance profiles of our isolates with other Cerro and Schwarzengrund strains available in NCBI, we re-analyzed publicly available genomes using Resfinder and summarized the antibiotic-specific resistance data in Figure 1. In this bar graph, the *aac(6′)-Iaa_1* gene, conferring resistance to the aminoglycosides amikacin (AMI) and tobramycin (TOB), was present in 100% of both serotypes. For antibiotics outside the aminoglycosides class, *S.* Cerro exhibited resistance frequencies of approximately 10–40% across the agents evaluated. In contrast, *S.* Schwarzengrund displayed higher resistance rates (20–40%) for most antibiotic genes and a notably high frequency (~85%) of ciprofloxacin (CIP) resistance, primarily due to the presence of the *qnrB19_1* gene.

**Figure 2:**
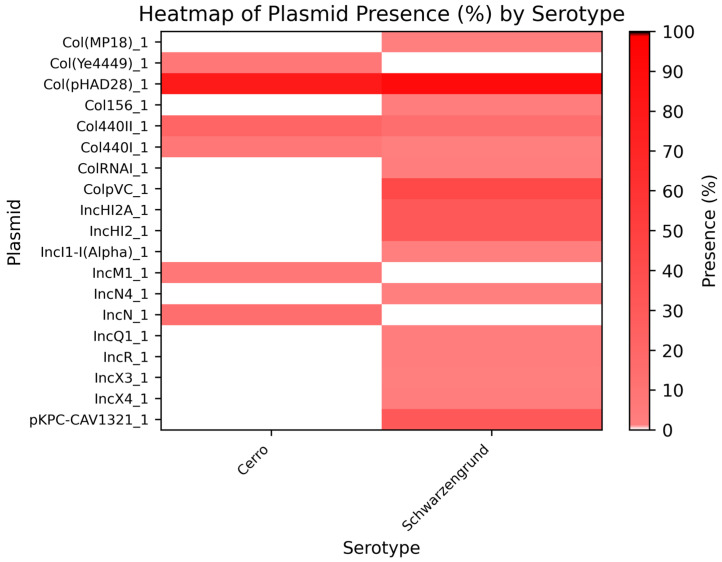
Heatmap of plasmid prevalence in *Salmonella* Cerro and Schwarzengrund. Legend: Heatmap indicates the presence (%) of plasmids in Cerro and Schwarzengrund. The color gradient ranges from white (0% presence) to black (100% presence).

Regarding plasmids, we conducted a comparative genomic annotation of Brazilian genomes from the Cerro and Schwarzengrund serovars and observed greater genomic plasticity in Schwarzengrund. This finding aligns with our isolates, where the *Col440I_1* plasmid was detected exclusively in this serovar. Although the overall frequency of *Col440I_1* was similar between Cerro and Schwarzengrund, other plasmids, such as *Col* (pHAD28), were more frequently identified in both serovars, despite being absent in our isolates (Figure 2). The *Col440I_1* plasmid belongs to the *ColE* family and functions as a cryptic plasmid involved in initiating plasmid DNA replication within the host cell [37]. In contrast, *Col* (pHAD28) has been described as originating from *Klebsiella pneumoniae* and is widely implicated in the dissemination of aminoglycoside resistance, often in association with genes from the *aac(6′)* family [38].

### 3.3. Phylogeny of Salmonella Cerro and Schwarzengrund and SPI Determinants

**Figure 3:**
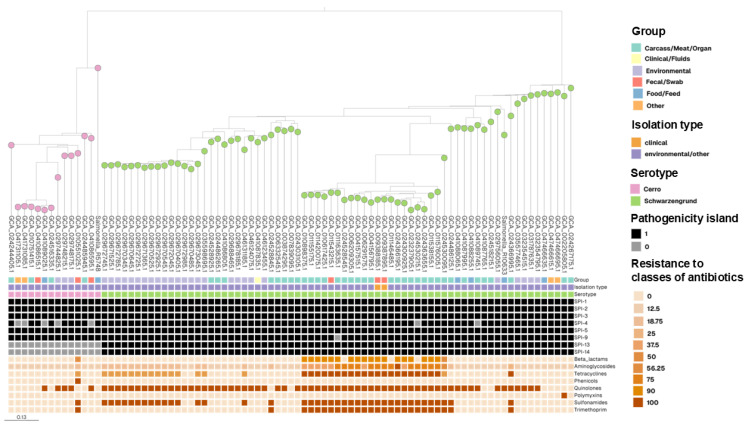
Phylogenetic tree of sequenced *Salmonella* Cerro and *Salmonella* Schwarzengrund serotypes present in the NCBI database, their virulence and frequency of antibiotic classes. Legend: The strains sequenced in this study are named *Salmonella*_R5748 and *Salmonella*_R10633. Beta_lactams: Amoxicillin, Ampicillin, Aztreonam, Cefepime, Cefotaxime, Ceftazidime, Ceftriaxone, Cephalothin, Piperacillin, Ticarcillin. Aminoglycosides: Amikacin, Apramycin, Gentamicin, Kanamycin, Neomycin, Netilmicin, Paromomycin, Tobramycin, Streptomycin, Dibekacin, Ribostamycin, Sisomicin, Spectinomycin, Hygromycin, Lividomycin. Tetracyclines: Tetracycline, Doxycycline, Minocycline. Phenicols: Chloramphenicol, Florfenicol. Quinolones: Ciprofloxacin. Polymyxins: Colistin. Sulfonamides: Sulfamethoxazole. Trimethoprim: Trimethoprim. Pathogenicity island: (1) Present, (0) Absent.

A total of 3493 *Salmonella* genomes were downloaded from the NCBI database, from which all the available isolates belonging to the Cerro and Schwarzengrund serovars were identified. To infer their evolutionary relationships, we constructed a maximum-likelihood phylogenetic tree based on the core genome, incorporating 85 genomes (71 Schwarzengrund and 14 Cerro) (Figure 3). In this tree, isolates from the Cerro and Schwarzengrund serovars form distinct clades that correlate with serotype, source and isolation type. Branch annotations further indicate the presence or absence of key virulence genes located within *Salmonella* pathogenicity islands (SPIs), as highlighted in the figure.

The clades observed in this study clearly demonstrate the separation between the Cerro and Schwarzengrund serovars, particularly in relation to pathogenicity islands. All Cerro isolates lacked islands 13 and 14, whereas these islands were consistently present in Schwarzengrund strains (Figure 3). In addition, a higher overall resistance to antibiotic classes was observed in Schwarzengrund compared to Cerro strain, which may partially explain the greater number of sequenced genomes available for this serovar.

Regarding the strains sequenced in this study, *Salmonella*_R5748 (Cerro) showed a notable phylogenetic distance from the other Cerro isolates available in the NCBI database. Although the reference strains are of the same geographical origin (Brazil) and have a similar antimicrobial resistance profile (low resistance to antibiotic classes), hypotheses may explain this phylogenetic distance, such as the origin of contamination. The Cerro strain in this study was isolated from food, while the others were isolated from other contaminated matrices; only one strain (GCA_024516335.1) had the same contaminated origin (food), but it was phylogenetically distant. In contrast, *Salmonella*_R10633 (Schwarzengrund) clustered closely with an environmentally derived isolate (GCA_029756055.1), sharing a similar profile in terms of pathogenicity island content and antibiotic resistance patterns.

**Figure 4:**
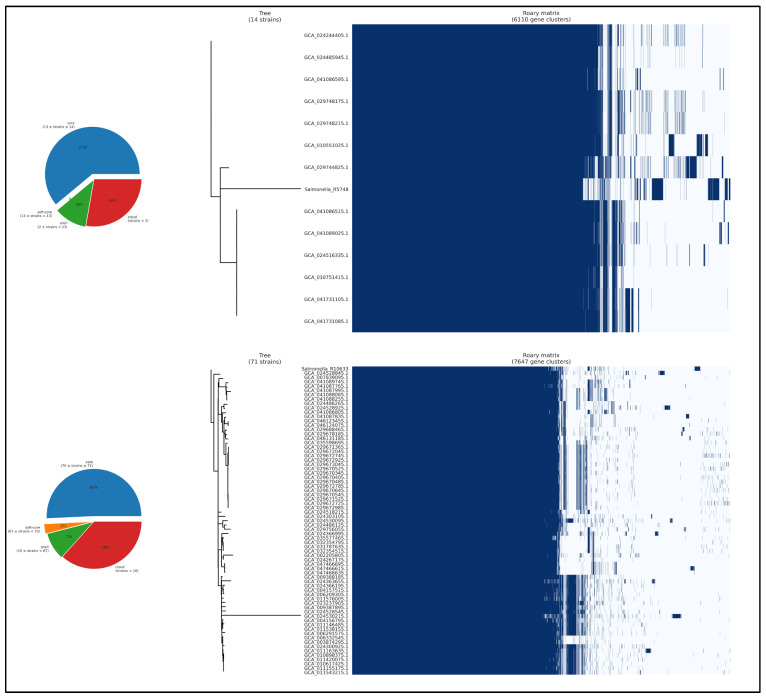
Pangenome composition and core-genome SNP phylogeny of *Salmonella* Cerro and Schwarzengrund isolates. Legend: The figures on the left show a pie chart of pangenome composition, dividing gene clusters into core (present in >99% of strains; in blue), soft core (95–99%; orange), shell (15–95%; green) and cloud (<15%; red). On the right, a core-genome SNP phylogeny (dendrogram) is displayed alongside a binary presence/absence matrix (blue = gene present; white = gene absent).

In Figure 4, the pangenome of *Salmonella* Cerro (14 strains) and *S.* Schwarzengrund (71 strains) is contrasted. In *S.* Cerro, 3730 gene clusters are shared by more than 99% of genomes (core genome), with no clusters identified in the soft core. Also, 685 clusters belong to the shell, and 1695 are found in less than 15% of genomes (cloud). For *S.* Schwarzengrund, 3879 clusters constitute the core, 251 the soft core, 716 the shell and 2801 the cloud. The core-genome SNP phylogeny reveals two major subclades within the Cerro lineage and at least three well-supported lineages in Schwarzengrund. The adjacent presence/absence heatmap illustrates that the patterns of accessory gene gain and loss closely correspond to the phylogenetic clades, suggesting that differential acquisition of accessory genes contributes to lineage divergence and may reflect specific adaptative processes.

### 3.4. Salmonella Pathogenicity Islands and Virulence Genes

**Figure 5:**
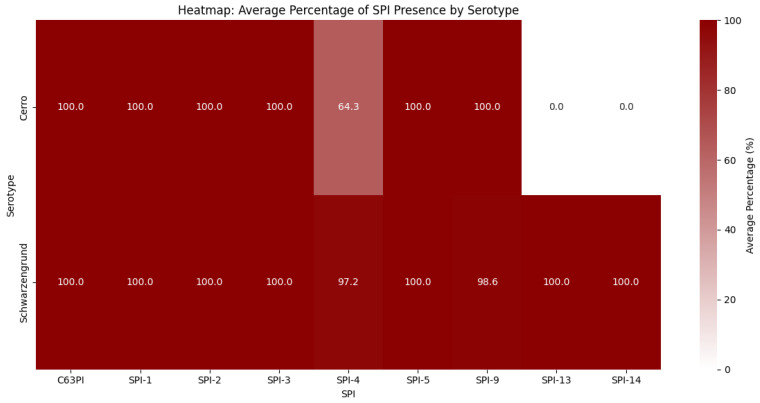
Heatmap of the frequency of SPIs in relation to serovars. Legend: Heatmap showing the frequency of pathogenicity islands (SPIs) under the serotypes in shades of red.

Expanding the analysis of pathogenicity islands, Figure 5 shows that there is a considerable difference between the two serovars, especially in the presence of SPI-4, SPI-13 and SPI-14. These islands are mainly associated with key virulence functions: SPI-4 contributes to host cell adherence and cytotoxicity; SPI-13 is involved in macrophage internalization and intracellular survival; and SPI-14 plays a role in systemic virulence and bile salt tolerance, facilitating survival during passage through the gastrointestinal tract and subsequent invasion of internal organs.

**Figure 6:**
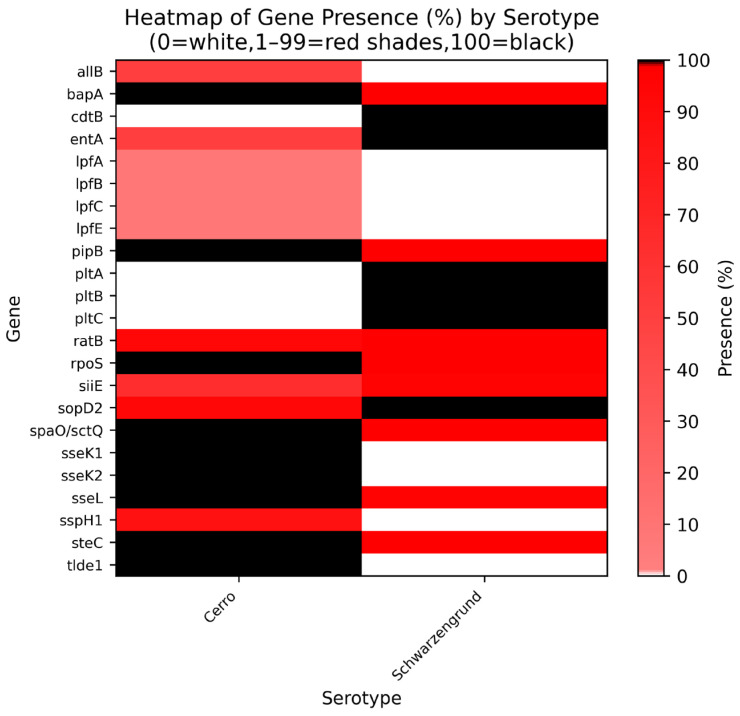
Heatmap of the percentage presence of virulence genes in *Salmonella* Cerro and Schwarzengrund isolates. Legend: Heatmap of the percentage presence of selected virulence genes in isolates of *Salmonella* serotypes Cerro and Schwarzengrund. The vertical axis lists the genes analyzed; the horizontal axis lists the serotypes. The color gradient ranges from white (0% presence) to black (100% presence). *Salmonella*_R5748 (Cerro) demonstrated the presence of the following genes: *allB*, *bapA*, *entA*, *lpfA*, *lpfB*, *lpfC*, *lpfE*, *pipB*, *rpoS*, *siiE*, *spaO/sctQ*, *sseK1*, *sseK2*, *sseL*, *steC tlde1*, while exhibiting absence of the following genes: *cdtB*, *pltA*, *pltB*, *pltC*, *ratB*, *sopD2*, *sspH1. Salmonella*_R10633 (Schwarzengrund) demonstrated the presence of the following genes: *bapA*, *cdtB*, *entA*, *pipB*, *pltA*, *pltB*, *pltC*, *ratB*, *rpoS*, *siiE*, *sopD2*, *spaO/sctQ*, *sseK1*, *sspH1*, *tlde1*, while exhibiting absence of the following genes: *allB*, *lpfA*, *lpfB*, *lpfC*, *lpfE*, *sseK2*, *sseL*, *steC*.

Finally, we assessed the virulence gene profiles of Cerro and Schwarzengrund isolates to identify potential differences between the groups. Genomic annotation using the VFDB database revealed a total of 139 virulence genes across all genomes. Among these, 116 genes were conserved across 100% of the isolates, while only 23 genes showed variation between the two serovars (Figure 6).

The results shown in Figure 6 indicate the presence of genes from the *pltABC* family in all Schwarzengrund genomes, while none of the Cerro strains carried these genes. The *pltABC* gene cluster encodes a tripartite exotoxin, originally identified in *Salmonella typhi*, which contributes to host cell damage and disruption of the immune response. Similarly, the *cdtB* gene was detected exclusively in Schwarzengrund isolates. On the other hand, *S.* Cerro genomes consistently harbored the *sseK1* and *sseK2* genes (responsible for favoring intracellular survival and systemic dissemination), as well as the *tlde1* gene (responsible for encoding a toxin with an antimicrobial effect on competing bacterial cells).

## 4. Discussion

### 4.1. Serovar Determination and Sequence Classification

Serotyping via agglutination remains the initial step in the characterization of *Salmonella* [39], although in silico platforms based on WGS are increasingly employed for faster and more reliable serotype prediction [40]. In the present study, we observed discrepancy between traditional serology and WGS for *Salmonella*_R5748, which was initially classified as Schwarzengrund via serology but later confirmed as Cerro by SeqSero. According to [41], an agreement rate was observed between traditional serotyping and WGS using SeqSero; however, 7.7% of the isolates showed discordant results, and 5.9% remained untyped by the molecular tool. Thus, these findings underscore the transformative potential of WGS-based tools in public health microbiology [40,42]. The current global trend is to use whole-genome sequencing in outbreak detection and routine surveillance of pathogenic bacteria [43]. Based on the studies highlighting the importance of WGS, it is valuable to emphasize that this tool could easily make a significant contribution to surveillance systems in Brazil.

*Salmonella* Cerro and Schwarzengrund have been reported worldwide across a variety of food matrices [6,9,44,45], and dozens of isolates have had their genomes sequenced and made publicly available in the NCBI database. In contrast, only a limited number of studies in Brazil have performed WGS to characterize *Salmonella* isolates [13,15]. To our knowledge, this is the first study is Mato Grosso to apply WGS for the characterization of *Salmonella* serotypes isolated from both cheese and beef, thereby addressing a critical gap in regional surveillance and food safety research.

*S.* Schwarzengrund is a relatively uncommon cause of human salmonellosis worldwide [46], but its incidence has increased in recent decades in countries such as Thailand, Slovakia, New Zealand and Venezuela [46]. Its prevalence has also risen in Japan, where isolates have been recovered from chicken meat [46,47]. In China, poultry meat remains the primary source of contamination, along with cases in humans and animals [48]. Reflecting these trends, nearly half (49.3%) of all *S.* Schwarzengrund genomes in the NCBI Pathogen Detection database originate from carcasses, meat and organs. In the present study, we identified that there is a global convergence regarding the source of isolation that has been reported in the literature with our findings, since the sequenced Schwarzengrund strain was isolated from beef.

Poultry, especially chicken, has been identified as the main vehicle for *S.* Schwarzengrund infections [49,50]. In Brazil, *S.* Schwarzengrund has similarly been isolated from poultry and chicken meat [51,52], and more recently from beef [24], highlighting its relevance across multiple food matrices.

*S.* Cerro is rarely implicated in human salmonellosis [53], although it has been associated with clinical disease in cattle in the United States [54]. In Brazil, *S.* Cerro has been recovered from poultry [55], swine [56] and wild boar [57]. Despite this diversity of hosts, its prevalence in Brazilian cattle remains poorly characterized; however, one study did report the detection of *S.* Cerro in veal calves [58]. The relatively low incidence of human infection caused by *S.* Cerro, especially compared to *S.* Schwarzengrund, likely contributes to its under-representation in the NCBI Pathogen Detection database.

*Salmonella* primarily colonizes the intestinal tract of humans and animals, including cattle [59]. Salmonellosis in cattle is a global concern, with serotypes such as *S*. *Dublin* and *S*. *Typhimurium* commonly implicated [60]. Notably, outbreaks linked to *Salmonella* transmission through raw milk in Denmark and raw meat in Ireland have been documented [61,62]. These incidents highlight the importance of ensuring food quality control measures to ensure consumer safety.

*Salmonella enterica* frequently colonizes both dairy products and livestock, serving as vehicles and reservoirs for human exposure [63,64]. Human salmonellosis is most commonly attributed to the consumption of contaminated poultry meat and eggs, as well as dairy and beef products [2]. In our metadata analysis, only 5.6% (4/71) of the *S.* Schwarzengrund isolates originated from food sources or animal feed, highlighting the under-representation of these reservoirs in publicly available genomic data.

*Salmonella* colonization in cattle can result in the contamination of both milk and meat at the farm level [65]. Cattle often carry the pathogen asymptomatically, introducing it into slaughterhouses and posing significant food safety risks through cross-contamination during processing. In this study, we focused on strains isolated from cheese and beef. Notably, no other *Salmonella* isolates from cheese were present in the current metadata, underscoring the novelty and relevance of our findings.

### 4.2. Detection of Resistance Genes and Plasmids in Salmonella Cerro and Schwarzengrund

Antimicrobial resistance can be transmitted to humans through the food chain, either via the ingestion of resistant bacteria or via horizontal gene transfer to other micro-organisms [66]. Resistance among foodborne pathogens represents a serious global public health threat [67,68]. In our study, we detected the *aac(6′)-Iaa_1* gene, associated with resistance to aminoglycosides, specifically amikacin (AMI) and tobramycin (TOB), in both sequenced serotypes. This same behavior was observed in reference strains available in the NCBI database, as both Cerro and Schwarzengrund harbored genes conferring resistance to this antibiotic class.

Although approximately 85 aminoglycoside-modifying enzymes have been identified, only a subset are commonly selected and responsible for the majority of aminoglycoside resistance. Among them, *aac(6′)-I* is particularly notable [69]. The same authors suggest that intrinsic resistance to aminoglycosides may be explained by the mechanism of antibiotic uptake, which depends on bacterial respiration. This process generates an electrochemical potential across the cytoplasmic membrane. However, a reduced or absent membrane potential, often observed in anaerobic bacteria, may account for resistance in these organisms [70].

The *qnrB19* gene was previously identified by [71] in the WGS of *Salmonella enterica* isolated from duck carcasses. Similarly, ref. [14] detected the *qnrB19* gene in serotypes circulating within the Brazilian food production chain. Their study was also the first in Brazil to report the presence of this gene in *S.* Schwarzengrund isolates from poultry and environmental samples. In addition, the *qnrB19* gene has been reported in other *Salmonella* serotypes in Brazil, with [72] documenting its presence in isolates from both food and human sources. In our phylogenetic analysis, we detected that most Schwarzengrund isolates have genes that confer resistance to the quinolone class (high frequency). In 2024, the World Health Organization [73] published a list of bacterial pathogens of public health importance, in which *Salmonella* Tiphi is in the high group category (difficult to treat and showing increasing trends of antimicrobial resistance), with an association of resistance to fluoroquinolones.

We observed that the *S.* Schwarzengrund serotype exhibited resistance to ciprofloxacin (CIP), a quinolone antibiotic, mediated by the *qnrB19_1* gene. Genes in the qnr family encode pentapeptide-repeat proteins that bind DNA gyrase, thereby blocking quinolone activity [13]. *Salmonella* spp. can serve as both recipients or donors of resistance determinants, facilitating horizontal gene transfer and posing significant food safety risks to human health [74]. Although CIP remains the drug of choice for treating complicated gastrointestinal infections, resistance among *Salmonella* isolates has been widely reported [75,76]. In addition, our findings reveal distinct resistome profiles for *S.* Cerro and *S.* Schwarzengrund: *S.* Cerro exhibited a lower frequency of antibiotic resistance genes across most antibiotic classes, while *S.* Schwarzengrund harbored a broader and more diverse resistome. Plasmids can play a significant role in antimicrobial resistance or virulence, with *Col440I_1* playing a role in initiating plasmid DNA replication within the host cell [37]. This leads us to hypothesize that they may be acting more directly in the relationship with MDR strains.

Mato Grosso ranked among Brazil’s top beef-exporting states in 2024, supplying predominantly China and the United States [12]. This highlights the critical importance of maintaining herd health to ensure the safety of animal-derived foods. However, the routine use of veterinary antibiotics (VAs) and synthetic growth promoters (SGPs) can compromise dietary sustainability and lead to the accumulation of residues in meat, milk and eggs [77]. Moreover, China (23%), the United States (13%), Brazil (13%), India (3%) and Germany (3%) together account for the highest levels of antimicrobial consumption in food animal production systems [78].

*Salmonella* gastroenteritis is typically treated with quinolones and fluoroquinolones, especially in elderly patients [79]. Ciprofloxacin is considered the drug of choice for salmonellosis and has been associated with the presence of the *qnrB19* gene in non-human isolates [80]. The *qnr* genes are typically plasmid-mediated, conferring resistance to quinolones, including ciprofloxacin, commonly used in veterinary medicine [81]. In our study, *S.* Schwarzengrund displayed elevated resistance across multiple antibiotics (Figure 1), with approximately 85% of the isolates resistant to CIP. The *qnrB19* gene was identified by [14] as the most prevalent resistance determinant in their study. Also, ref. [82] reported high levels of fluoroquinolone resistance among several serotypes, including *S.* Schwarzengrund. According to [83], such resistance may also result from mutations in the *gyrA* gene.

No plasmids were detected in the *S.* Cerro isolates in this study, whereas *S.* Schwarzengrund harbored a *Col440I_1* replicon. Although *Col440I_1*-type plasmids have been reported in both serotypes in other Brazilian isolates, our data demonstrate their presence exclusively in *S.* Schwarzengrund (Figure 2), highlighting their greater genomic plasticity. *Col440I_1* plasmids have been reported in *S.* Schwarzengrund by [22,84] in Chile. According to [85], plasmids can play a critical role in the antimicrobial resistance and virulence of *S.* Schwarzengrund. Consistent with these findings, we observed a higher prevalence of plasmids in *S.* Schwarzengrund compared to *S.* Cerro.

### 4.3. Phylogeny of Salmonella Cerro and Schwarzengrund and SPI Determinants

The phylogenetic analysis revealed a clear division between *Salmonella* Cerro and *Salmonella* Schwarzengrund, consistent with their classification as distinct serotypes. In Brazil, little is known about the occurrence of *S.* Cerro in cattle, and this serotype has not been reported in the literature as a direct cause of human salmonellosis [53]. Notably, within the *S.* Cerro clade, the strain sequenced in this study was phylogenetically more distant from the other Cerro isolates, a divergence further supported by the pangenome analysis. This distinct clustering may be partially explained by its limited antibiotic resistance (Figure 1), although a consistent resistance pattern across antibiotic classes was still observed among the Cerro strains.

According to [86], several studies have sought to explain why *S.* Cerro is frequently associated with livestock but rarely causes human disease. In the study by [53], the authors evaluated the diversity of *Salmonella* subtypes in dairy cattle and found that *S.* Cerro lacked virulence genes located in pathogenicity islands 10, 12 and 13. Consistent with these findings, Figure 5 shows that all *S.* Cerro genomes available in the NCBI database lack SPI-13 virulence genes, a pattern not observed in *S.* Schwarzengrund.

The clades and branches of the *S.* Schwarzengrund serotype form distinct isolation groups, with farm animals frequently identified as the sources of contamination. The isolate sequenced in this study did not cluster within any well-defined group but was phylogenetically closest to the GCA_029756055.1 strain, which grouped with strains recovered from environmental water. *S.* Schwarzengrund infections have increased globally in recent years [85]. Moreover, ref. [87] had documented infections caused by this serotype in both humans and birds across various Western countries. Notably, all strains clustering within our isolate were obtained in Brazil from poultry and environmental sources; none originated from beef.

Finally, upon comparison of the resistance frequencies across antibiotic classes, we observed that *S.* Schwarzengrund obtained from the NCBI database exhibited broad resistance profiles, with many strains classified as multidrug-resistant (Figure 3). In contrast, the strain sequenced in this study showed resistance only to the quinolone class. *S.* Cerro isolates, by comparison, displayed uniformly low resistance frequencies across all antibiotic classes.

### 4.4. Virulence Genes in Pathogenicity Islands

Pathogenicity islands are genomic regions acquired through horizontal gene transfer that contribute to microbial virulence [88]. Ref. [89] provided an early overview of their evolution in *Salmonella enterica* serotypes, initially describing twelve pathogenicity islands. Subsequent research has expanded this number to seventeen known islands in *Salmonella* [88,90]. Ref. [91] further elucidated how these islands enhance the pathogenic potential of different *Salmonella* serovars. In particular, ref. [92] identified several virulence genes in *Salmonella* serotypes that are implicated in transmission and infection via type III secretion system (T3SS), which plays a critical role in host invasion. The pathogenicity of *S.* Schwarzengrund is driven by a wide array of virulence factors, notably the T3SS, encoded by *Salmonella* islands I and II [85,93]. SPI-II activity is essential for the formation and maintenance of the *Salmonella*-containing vacuole (SCV), an intracellular niche that supports bacterial survival and replication [88].

*Salmonella* pathogenicity islands (SPIs) harbor a diverse array of virulence genes, most of which are chromosomally located [14]. These islands are horizontally acquired genomic segments that play a central role in *Salmonella* pathogenesis [94]. Of the approximately 23 SPIs described to date [95], SPIs 1 and 2 are considered the most critical virulence determinants in *Salmonella* [96].

Pathogenicity island I (SPI-1) is the most extensively characterized among the *Salmonella* pathogenicity islands [97]. According to [94], SPI-I encodes effector proteins that are secreted via type III secretion system, facilitating the invasion of intestinal epithelial cells. In contrast, SPI-2, SPI-3 and SPI-4 are primarily involved in promoting bacterial growth and survival within the host during the systemic phase of the infection [97].

*Salmonella* pathogenicity island 4 (SPI-4) encodes six main genes: *sii*A–F. Among them, *Sii*C, *Sii*D and *Sii*F form a type I secretion system responsible for secreting *Sii*E [88]. In our study, *Sii*E was detected in all sequences analyzed. This gene mediates adhesion to bovine intestinal epithelial cells [98]. Also, genes of the *plt*ABC operon encode an exotoxin that damages host cells and subverts immune defenses [99]. These virulence genes were present in our *S.* Schwarzengrund isolate but were absent from all *S.* Cerro strains examined.

In *S.* Cerro, the effector genes *sse*K1 and *sse*K2 were detected in 100% of the isolates and were absent from all *S.* Schwarzengrund strains. These genes are located on *Salmonella* pathogenicity island II [100].

Until recently, the contributions of the *sse*K2 gene to *Salmonella* virulence has not been well understood. Refs. [99,100] examined the role of *sse*K2 in *Salmonella enterica* and demonstrated that deletion of this gene significantly attenuates pathogenicity both in vivo and in vitro. *Sse*K2 is a novel translocated effector protein originally described in *S. typhimurium* and is highly conserved across *Salmonella* strains [100].

We observed that Cerro lacked virulence genes located on pathogenicity islands 13 and 14. Similarly, ref. [53] reported the absence of SPI-13 in *S.* Cerro. The exact number of genes that constitute SPI-13 remains unclear [101,102]. This island has also been observed in other *S. enterica* subspecies [103,104], as well as in *S.* Gallinarum [105]. According to [106], an in silico analysis revealed that most genes within SPI-13 encode proteins involved in bacterial metabolism, leading to the hypothesis that the absence of this island may result in a nutritional disadvantage, potentially impairing *Salmonella* virulence in specific hosts.

In contrast, *S.* Schwarzengrund harbored genes located on pathogenicity islands 13 and 14, a finding also observed by [107]. Genes associated with SPI-14 play a central role in the activation of SPI-1 genes, primarily through a regulator encoded within SPI-14 (STM14_1008) known as low-oxygen-induced factor A (*LoiA*), which functions as a key virulence determinant [108].

### 4.5. Limitations

Although the data obtained for the *Salmonella* Cerro and *Salmonella* Schwarzengrund serovars are relevant, it is important to acknowledge some limitations. Given the number of strains sequenced (N = 2) and the lack of phenotypic validation, the observed percentages of Cerro and Schwarzengrund strains may not accurately reflect their true prevalence due to potential selection bias in sequencing efforts. Serotypes such as *S*. Typhimurium, *S*. Typhi and *S*. Enteritidis are more frequently studied and sequenced, which does not necessarily imply that they are more prevalent in the environment than Cerro and Schwarzengrund. We encourage further studies focusing on these less characterized serotypes, as the findings related to resistance genes, virulence factors, plasmid presence and pathogenicity islands presented here represent potential capabilities rather than definitive resistance or virulence profiles.

Finally, it is important to note that the strains sequenced in this study were isolated from food sources, which may affect comparisons with clinical isolates in public databases. Clinical strains are generally better characterized and tend to exhibit higher resistance profiles than environmental or food-derived isolates.

## 5. Conclusions

In conclusion, *Salmonella* Cerro and *Salmonella* Schwarzengrund represent a small fraction of the strains isolated and sequenced in the NCBI database. Our analysis revealed that *S.* Cerro exhibited a lower antimicrobial resistance profile but carried virulence gene clusters with potential threat to human health. Notably, the *S.* Cerro virulome lacked genes located on SPI-13 and SPI-14, whereas *S.* Schwarzengrund harbored genes associated with both islands. A virulome analysis indicated that most *S.* Schwarzengrund strains were multidrug-resistant (MDR); however, the strain sequenced in this study showed resistance only to aminoglycoside and quinolone. Although only one plasmid was detected in our *S.* Schwarzengrund isolate, a broader diversity of plasmids was observed in both serotypes across the dataset, underscoring the potential for future dissemination of resistance and virulence genes through horizontal gene transfer.

## Figures and Tables

**Table 1:** Resistance genes and plasmids found in the sequenced serotypes through WGS.

Strain	*S.* Cerro (*Salmonella*_R5748)	*S.* Schwarzengrund (*Salmonella*_R10633)	Classes of Antibiotics/Function
**Resistance genes**	*Aac(6′)-Iaa_1*	*Aac(6′)-Iaa_1* *qnrB19_1*	Aminoglicosides Quinolones
**Plasmids**	None	*Col440I_1*	They play a role in antimicrobial resistance and virulence

## Data Availability

The original contributions presented in this study are included in the article and Supplementary Materials. Further inquiries can be directed to the corresponding author.

## Supplementary Materials

The following supporting information can be downloaded at: https://www.mdpi.com/article/10.3390/genes16080880/s1, Table S1: Quality of sequences.
