# Supplementary material for: Integrated Whole-Genome Sequencing and In Silico Characterization of Salmonella Cerro and Schwarzengrund from Brazil
Source: Genes (Basel). 2025 Jul 26;16(8):880. doi: 10.3390/genes16080880 (PMC12385404; doi:10.3390/genes16080880)
Supplement: Supplementary file 1 [file genes-16-00880-s001.zip › genes-3746477-Supplementary table.pdf]

Supplementary Table S1: Quality of sequences.

#File	num_seqs	sum_len	min_len	avg_len	max_len	N50	N50_num	GC(%)
GCA_024530215.1_PDT001372450.1_genomic.fna	388	4940618	389	12733.6	96190	20764	68	51.76
GCA_024363655.1_PDT001364895.1_genomic.fna	285	4859949	386	17052.5	175923	34588	40	51.77
GCA_004156795.1_PDT000316794.1_genomic.fna	133	4878757	462	36682.4	232325	78441	19	51.87
GCA_011576005.1_PDT000309444.2_genomic.fna	125	4893700	462	39149.6	276893	92392	17	51.89
GCA_041731105.1_ASM4173110v1_genomic.fna	71	4617130	709	65030.0	382577	108768	14	52.28
GCA_041087765.1_ASM4108776v1_genomic.fna	71	4647888	418	65463.2	284272	122459	13	51.98
GCA_041089025.1_ASM4108902v1_genomic.fna	87	4636831	417	53296.9	383237	102359	13	52.22
GCA_011155175.1_PDT000309420.2_genomic.fna	97	4879937	462	50308.6	511791	120406	12	51.87
GCA_011538155.1_PDT000304890.2_genomic.fna	85	4908566	456	57747.8	471375	139033	12	51.82
GCA_006209305.1_PDT000316830.1_genomic.fna	107	4902355	462	45816.4	296809	126659	11	51.84
GCA_011420075.1_PDT000312232.2_genomic.fna	73	4865999	462	66657.5	329383	150830	11	51.87
GCA_024528545.1_PDT001372438.1_genomic.fna	80	4900481	406	61256.0	426243	175198	11	51.83
GCA_041086515.1_ASM4108651v1_genomic.fna	66	4593431	321	69597.4	382725	158603	10	52.26
GCA_041087835.1_ASM4108783v1_genomic.fna	47	4649103	406	98917.1	505169	170834	10	52.14
GCA_004157515.1_PDT000316789.1_genomic.fna	84	4910437	462	58457.6	545700	189129	9	51.83
GCA_006291575.1_PDT000316780.1_genomic.fna	83	4912618	442	59188.2	657061	153271	9	51.82
GCA_006332545.1_PDT000316791.1_genomic.fna	55	4601187	482	83657.9	506143	154107	9	52.15
GCA_024528845.1_PDT001372473.1_genomic.fna	71	4755742	406	66982.3	491966	211150	9	52.05

GCA_041086595.1_ASM4108659v1_genomic.fna	52	4492883	481	86401.6	395171	163240	9	52.34
GCA_041088065.1_ASM4108806v1_genomic.fna	46	4709248	370	102375.0	386759	182429	9	51.94
GCA_041731085.1_ASM4173108v1_genomic.fna	57	4621138	639	81072.6	491952	175531	9	52.28
GCA_010551025.1_PDT000177749.2_genomic.fna	56	4704065	432	84001.2	420535	209479	8	52.24
GCA_010898375.1_PDT000304969.2_genomic.fna	72	4861474	462	67520.5	597313	198759	8	51.88
GCA_024366195.1_PDT001365221.1_genomic.fna	66	4897582	462	74205.8	412371	234305	8	51.81
GCA_024366995.1_PDT001365179.1_genomic.fna	91	4755423	387	52257.4	517222	214204	8	51.91
GCA_024516335.1_PDT001374826.1_genomic.fna	45	4615676	398	102570.6	450017	214398	8	52.26
GCA_024530095.1_PDT001372472.1_genomic.fna	73	4850867	418	66450.2	467934	208216	8	51.9
GCA_029744825.1_PDT001689645.1_genomic.fna	57	4742145	335	83195.5	385149	193852	8	52.21
GCA_041088255.1_ASM4108825v1_genomic.fna	44	4711870	462	107088.0	368207	218645	8	51.94
GCA_041089745.1_ASM4108974v1_genomic.fna	53	4658319	462	87892.8	351848	214151	8	51.99
GCA_010617425.1_PDT000312230.2_genomic.fna	68	4868455	450	71594.9	815146	188900	7	51.87
GCA_010751415.1_PDT000177747.2_genomic.fna	44	4541960	544	103226.4	542171	194018	7	52.32
GCA_011146485.1_PDT000304910.2_genomic.fna	60	4902961	462	81716.0	739229	219082	7	51.81
GCA_011163635.1_PDT000309428.2_genomic.fna	73	4962009	462	67972.7	695149	214418	7	51.82

GCA_023237905.1_PDT001298917.1_genomic.fna	68	4953521	462	72845.9	509708	315651	7	51.79
GCA_024244405.1_PDT001358076.1_genomic.fna	40	4569059	481	114226.5	500678	232424	7	52.26
GCA_024300925.1_PDT001362070.1_genomic.fna	63	4904144	420	77843.6	467931	234536	7	51.8
GCA_024485945.1_PDT001372468.1_genomic.fna	43	4469312	392	103937.5	508087	221652	7	52.37
GCA_024486125.1_PDT001372445.1_genomic.fna	49	4639139	422	94676.3	404167	224638	7	52.07
GCA_024518215.1_PDT001374705.1_genomic.fna	35	4671838	461	133481.1	443406	278353	7	51.99
GCA_046131185.1_PDT002538622.1_genomic.fna	54	4650684	227	86123.8	441377	278098	7	52.17
GCA_024267175.1_PDT001358676.1_genomic.fna	36	4567537	462	126876.0	468373	339440	6	52.14
GCA_024303105.1_PDT001362074.1_genomic.fna	35	4658327	462	133095.1	514687	366756	6	52.06
GCA_024486265.1_PDT001372406.1_genomic.fna	40	4633928	451	115848.2	467574	264967	6	52.18
GCA_024528925.1_PDT001372440.1_genomic.fna	47	4735810	461	100761.9	868671	234570	6	52.02
GCA_029748175.1_PDT001689772.1_genomic.fna	57	4653906	309	81647.5	527981	312169	6	52.23
GCA_029748215.1_PDT001689776.1_genomic.fna	54	4653456	362	86175.1	533538	312571	6	52.23
GCA_041087995.1_ASM4108799v1_genomic.fna	46	4710643	471	102405.3	652066	225927	6	51.94
GCA_046123455.1_PDT002539179.1_genomic.fna	51	4642745	227	91034.2	767091	224831	6	52.15
GCA_046124075.1_PDT002539146.1_genomic.fna	51	4625660	227	90699.2	525682	278098	6	52.16

GCA_011543215.1_PDT000304986.2_genomic.fna	56	4873921	462	87034.3	1154611	327115	5	51.85
GCA_041086805.1_ASM4108680v1_genomic.fna	39	4675495	370	119884.5	1135488	265198	5	52.01
GCA_002205805.1_ASM220580v1_genomic.fna	23	4621439	1729	200932.1	995476	412101	4	52.04
GCA_003874295.1_PDT000304892.2_genomic.fna	33	4611668	462	139747.5	673311	441048	4	52.13
GCA_029670405.1_PDT001686506.1_genomic.fna	40	4755442	482	118886.1	803811	424810	4	52.12
GCA_029670485.1_PDT001686492.1_genomic.fna	31	4758428	487	153497.7	1182732	398206	4	52.12
GCA_029672925.1_PDT001686404.1_genomic.fna	34	4745462	462	139572.4	1109252	412740	4	52.16
GCA_029673045.1_PDT001686399.1_genomic.fna	38	4767224	370	125453.3	1114259	389935	4	52.13
GCA_035577465.1_PDT002050695.1_genomic.fna	31	4633590	373	149470.6	1145109	397547	4	52.03
GCA_007839095.1_PDT000177726.2_genomic.fna	25	4606144	462	184245.8	1480315	390107	3	52.14
GCA_029670345.1_PDT001686508.1_genomic.fna	33	4715596	462	142896.8	1525297	468465	3	52.18
GCA_029670525.1_PDT001686490.1_genomic.fna	35	4796814	462	137051.8	1524820	468465	3	52.13
GCA_029670545.1_PDT001686477.1_genomic.fna	34	4731437	482	139159.9	1482721	468465	3	52.17
GCA_029670645.1_PDT001686468.1_genomic.fna	35	4774982	370	136428.1	1524995	468465	3	52.12
GCA_029671365.1_PDT001686494.1_genomic.fna	34	4749872	487	139702.1	1433490	469517	3	52.16
GCA_029671525.1_PDT001686480.1_genomic.fna	34	4743498	406	139514.6	1524636	468465	3	52.16

GCA_029672045.1_PDT001686447.1_genomic.fna	39	4730037	406	121283.0	1525289	397984	3	52.16
GCA_029672725.1_PDT001686413.1_genomic.fna	34	4821101	406	141797.1	1524826	468465	3	52.11
GCA_029672745.1_PDT001686412.1_genomic.fna	36	4797043	370	133251.2	1525289	441624	3	52.12
GCA_029672785.1_PDT001686411.1_genomic.fna	31	4768148	462	153811.2	1524822	468465	3	52.14
GCA_029672985.1_PDT001686400.1_genomic.fna	30	4723535	462	157451.2	1524814	468465	3	52.12
GCA_029678185.1_PDT001686396.1_genomic.fna	31	4760849	406	153575.8	1490933	468355	3	52.12
GCA_029688465.1_PDT001687398.1_genomic.fna	32	4722917	482	147591.2	1493954	467174	3	52.14
GCA_029756055.1_PDT001689885.1_genomic.fna	31	4736711	370	152797.1	1511417	435678	3	51.98
GCA_031787635.1_PDT001829414.4_genomic.fna	29	4668421	482	160980.0	1504354	441624	3	52.02
GCA_032354795.1_PDT001829449.1_genomic.fna	27	4626578	528	171354.7	1503893	398206	3	52.03
GCA_035598695.1_PDT002050399.1_genomic.fna	35	4690356	332	134010.2	1480315	424819	3	52.2
GCA_047466615.1_ASM4746661v1_genomic.fna	39	4670511	299	119756.7	1552125	517423	3	51.93
GCA_047466635.1_ASM4746663v1_genomic.fna	44	4612098	249	104820.4	1552271	461929	3	52.12
GCA_047466695.1_ASM4746669v1_genomic.fna	34	4606521	283	135485.9	1471500	517472	3	52.13
GCA_032354515.1_PDT001829475.1_genomic.fna	27	4665515	482	172796.9	1899520	694492	2	52.01
GCA_009387895.1_ASM938789v1_genomic.fna	3	4909930	2989	1636643.3	4630202	4630202	1	51.83

GCA_009388185.1_ASM938818v1_genomic.fna	3	4911019	2989	1637006.3	4631209	4631209	1	51.83

Legend: This table provides detailed information on the NCBI contig assemblies.
